# Monotonic load datasets for additively manufactured thermoplastic reinforced composites

**DOI:** 10.1016/j.dib.2020.105295

**Published:** 2020-02-19

**Authors:** Octavio Andrés González-Estrada, Alberto David Pertuz Comas, Jorge Guillermo Díaz Rodríguez

**Affiliations:** GIEMA, School of Mechanical Engineering, Universidad Industrial de Santander, Carrera 27 Calle 9, Bucaramanga, Colombia

**Keywords:** Additive manufacturing (AM), Fused deposition modeling (FDM), Continuous fiber reinforced thermoplastic composites (CFRTPC), Stress in thermoplastics, Mechanical properties

## Abstract

In additive manufacturing (AM), thermoplastic components made by fused deposition modeling (FDM) offer low strength and stiffness, as required for fully functional and load-bearing parts. Composite materials are a practical solution to improve mechanical properties [1,2]. A new technology to reinforce thermoplastics with continuous fibers has been developed recently by Markforged [3]. It introduces continuous fiber to reinforce a thermoplastic matrix, thus, taking static mechanical performance close to Aluminum alloys [4]. These printers for continuous fiber reinforced thermoplastic composites (CFRTPC) have taken this technology to a whole new level in terms of mechanical properties and efficient production. Mechanical properties under monotonic load were studied for different kinds of printing configurations. Tensile monotonic tests under controlled displacement were performed until rupture. Raw data showing tensile monotonic behavior provides the researchers with the ability to perform data fitting, to validate more advanced constitutive models, or to perform a further interpretation of the data, among others. Data is presented here as plain text files without any analysis. A preliminary data analysis has been published already in [5]. The text files contain information about time, displacement, and force. The data is useful for design engineers and researchers involved with AM.

**Table:** Specifications Table

Subject	Engineering/Mechanical Engineering
Specific subject area	Monotonic dataset for 3D printed composites samples reinforced with long continuous fibers.
Type of data	Plain text files.
How data were acquired	Instruments: MTS Bionix 370.02 Universal Testing Machine with a 25 kN load cell, and laser extensometer LX500. MTS suite software for displacement control and data acquisition.
Data format	Raw.
Parameters for data collection	All tests were conducted at controlled displacement. Ambient temperature (24 C). Sampling frequency of 20 Hz.
Description of data collection	Data was collected from zero loading until the sample was fully ruptured, except for the specimens printed without fiber reinforcement in which case, the test was interrupted when the displacement reached testing limits. Acquired variables are: load, displacement by the laser extensometer, displacement by the machine, number of cycles, and testing time.
Data source location	Institution: Universidad Industrial de Santander Bucaramanga/Santander Colombia:
Data accessibility	https://doi.org/10.17632/jj5jzww3vp.1 as Mendeley data set
Related research article	Alberto D. Pertuz, Sergio Díaz-Cardona, Octavio Andrés González-Estrada. Static and fatigue behavior of continuous fiber reinforced thermoplastic composites manufactured by fused deposition modeling technique. International Journal of Fatigue (13) 2020. https://doi.org/10.1016/j.ijfatigue.2019.105275 [5]

**Table:** 

**Value of the Data** • The raw data is useful in further research to estimate the optimal filling pattern and the linear elastic behavior assumed in the associated publication. Data could be used to perform data fitting, to validate more advanced constitutive models, or to perform a further interpretation of the data. Furthermore, the data can be used for reproducibility of the experiments or by simulation or theoretical researchers who need test data to feed their models. • The data is of interest to people involved in the testing, design, modeling, and manufacture of parts and components made of CFRTPC. • These data can be used for: validation of constitutive models specifically tailored for CFRTPC, including printing variables, for widening the sampling of CFRTPC, such that a lower uncertainty of mechanical properties can be established. Moreover, the data could be used as part of a machine learning and data analytics study to further learn about the mechanical behavior of CFRTPC. • The data provides a time frame in the interaction of displacement and force for CFRTPC under monotonic loading until rupture in the sample was detected. Additionally, the data provides an insight into how a hard fiber behaves in a soft polymer matrix.

## 1 Data description

Data sets files are presented in plain text format displaying the following columns: axial force (in N, and it is the applied force as given by the 25kN load cell attached to the MTS Bionix 370.02 machine), axial displacement (in mm, and it is the axial displacement as given by the LX500 laser extensometer), axial displacement (in mm, and it is the axial displacement as given by the MTS Bionix 370.02 machine's crosshead), axial count (number of applied loading cycles), and running time (in s as measured by the MTS suite software which controls the MTS Bionix 370.02 machine). The name and description of each file are shown in Table 1.

**Table 1:** Description of each provided file.

#	Filename	Description
1	FC 2A4C.txt	Carbon fiber reinforced with 2 concentric rings in a nylon matrix with 4 layers
2	FC 2C4A.txt	Carbon fiber reinforced with 4 concentric rings in a nylon matrix with 2 layers
3	FC 20%.txt	Carbon fiber with nylon matrix at 20% filling
4	FV60 20%.txt	Fiberglass reinforced with nylon matrix at 20% filling and 60° from the longitudinal axis
5	FV60f 20%.txt	Fiberglass reinforced with nylon matrix at 20% filling and 60° from the longitudinal axis
6	K0 20%.txt	Kevlar reinforced in nylon matrix at 20% filling and 0° from the longitudinal axis
7	FV0 20%.txt	Fiberglass reinforced with nylon matrix at 20% filling and 0° from the longitudinal axis
8	FV45 20%.txt	Fiberglass reinforced with nylon matrix at 20% filling and 45° from the longitudinal axis
9	Hex50.txt	Nylon with hexagonal 50% filling
10	Tri20.txt	Nylon with triangular 20% filling
11	Tri50.txt	Nylon with triangular 50% filling

Finally, it has to be highlighted that the files K020%.txt, FC20%.txt, FC 2A4C.txt, FC 2C4A.txt, FV60f 20%.txt do not have cross head axial displacement data and the FC 2A4C.txt, FC 2C4A.txt, FC20%.txt, FV60f 20%.txt files do not have cycle count data.

## 2 Experimental design, materials, and methods

Samples were made of composite materials reinforced with long fibers [1,2] and were manufactured using the Markforged Two printer [3,1,4] following ASTM D638-14 (Standard Test Method for Tensile Properties of Plastics) [6] using the dimensions of the type IV specimen. The tensile tests were conducted using displacement control, which was applied at a 5 mm/min rate.

The tests were conducted in the following manner. First, the matrix was tested to assess the performance of each filling (triangular at 20% filling, triangular at 50% filling, and hexagonal at 50% filling). Then, for the matrix filling at 20% and a triangular pattern, the fiber orientation was tested at 0°, 45° and 60° from the loading axis. The previous configurations with the three available fibers (Kevlar, carbon, and fiberglass) were tested. Finally, tests were done for two different carbon fiber reinforcement configurations using concentric rings: two rings with four layers, and four rings with two layers.
